# Complete genome of the onion pathogen *Enterobacter cloacae* EcWSU1

**DOI:** 10.4056/sigs.2174950

**Published:** 2011-12-22

**Authors:** Jodi L. Humann, Mark Wildung, Chun-Huai Cheng, Taein Lee, Jane E. Stewart, Jennifer C. Drew, Eric W. Triplett, Doreen Main, Brenda K. Schroeder

**Affiliations:** 1 Department of Plant Pathology, Washington State University, Pullman, WA, USA; 2 School of Molecular Biosciences, Washington State University, Pullman, WA, USA; 3 Department of Horticulture and Landscape Architecture, Washington State University, Pullman, WA, USA; 4 Department of Microbiology and Cell Science, University of Florida, Gainesville, FL, USA

Previous studies have shown that the members of the *Enterobacter cloacae* complex are difficult to differentiate with biochemical tests and in phylogenetic studies using multilocus sequence analysis, strains of the same species separate into numerous clusters. There are only a few complete *E. cloacae* genome sequences and very little knowledge about the mechanism of pathogenesis of *E. cloacae* on plants and humans. *Enterobacter cloacae* EcWSU1 causes Enterobacter bulb decay in stored onions (*Allium cepa*). The EcWSU1 genome consists of a 4,734,438 bp chromosome and a mega-plasmid of 63,653 bp. The chromosome has 4,632 protein coding regions, 83 tRNA sequences, and 8 rRNA operons.

## Introduction

*Enterobacter cloacae* is ubiquitous in nature and is known to cause disease in numerous plants, such as onion, ginger, papaya, and macadamia [1-4]. In addition, *E. cloacae* is an emerging opportunistic human pathogen that is associated with nosocomial infections [5]. Phylogenetic analyses of the genus *Enterobacter* have resulted in the formation of the *E. cloacae* complex, which consists of several species. The *E. cloacae* complex includes the species *E. cloacae,* *E. asburiae,* *E. hormaechei,* *E. kobei,* *E. ludwigii,* and *E. nimipressuralis*, but the list is constantly growing as new species of *Enterobacter* are identified. Within medical isolates of the *E. cloacae* complex, there are two well supported clades and 13 clusters [6]. The younger clade has less genetic diversity and is composed primarily of *E. hormaechei* strains isolated from hospitals. The second clade has more genetic diversity and contains the other members of the complex, including *E. cloacae*. Interestingly, *E. cloacae* strains separate into six clusters indicating considerable diversity within the species. A neighbor-joining tree of the *hsp60* gene from 206 *E. cloacae* strains showed that few *E. cloacae* strains (3%) actually cluster with the *type* strain, *E. cloacae* subsp. *cloacae* ATCC 13047 [7].

Enterobacter bulb decay develops after onions are harvested, cured, and stored. The decay usually occurs in a few scales of the onion bulb and the tissue develops a brown color giving the bulb a *dirty ring* appearance when cut in half [1,8]. If storage lots of onions have a high enough incidence of Enterobacter bulb decay (>2-5%), the whole lot cannot be sold and results in a significant loss to the grower. The mechanism of how *E. cloacae* causes bulb decay is unknown and as a result, the development of disease control methods for bulb decay are limited. In addition, many new strains are identified as *E. cloacae* due to traditional phenotype tests and 16S rRNA identity, but when other regions of the genome, or the genome as a whole, are compared, they appear to have more differences within a species than observed between species of other genera of bacteria [6, Humann and Schroeder, unpublished]. The genome sequence reported here will allow for comparisons on a genome-wide level with other *E. cloacae* strains and may help clarify the relationships between the *E. cloacae* complex members as well as allow for identification of putative pathogenesis genes.

## Classification and features

**Figure 1:**
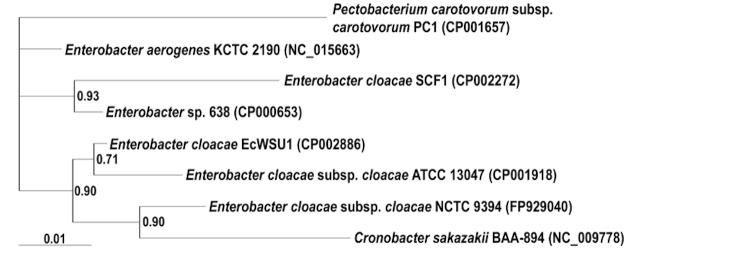
Phylogenetic tree of 16S rRNA sequences from strains of *Enterobacter* with genome sequences. Bayesian phylogenetic analyses of the 16S rRNA region yielded two distinct clusters, supported with a 0.90 posterior probability. Analyses were implemented in MRBAYES [24]. The Bayesian Information Criterion (BIC), DT-ModSel [25] was used to determine the nucleotide substitution model best suited for the dataset. The Markov chain Monte Carlo search included two runs with four chains each for 1,000,000 generations, ensuring that the average split frequencies between the runs was less than 1%. *Pectobacterium* served as the outgroup for the analysis. Numbers in parentheses behind the bacterial names correspond to the Genbank accession numbers for the genome sequences. The scale bar indicates the number of substitutions/site.

*E. cloacae* EcWSU1 was isolated from onion bulbs that were exhibiting symptoms of rot [8]. EcWSU1 is a Gram-negative, rod shaped bacterium of the family “*Enterobacteriaceae”* (Table 1). Species differentiation of the *Enterobacter* genus is difficult with biochemical and phylogenetic tests [6]. The genetic complexity of the *E. cloacae* complex is illustrated in a phylogenetic tree of the 16S rRNA region (Figure 1). EcWSU1 grouped with the type-strain *E. cloacae* subsp. *cloacae* ATCC 13047 with a 0.71 posterior probability in a Bayesian phylogenetic analysis. *E. cloacae* SCF1, isolated from soil in Puerto Rico, grouped closely with *Enterobacter* sp. 638 [26], an endophyte of poplar trees. *Cronobacter sakazakii* BAA-894, formerly *Enterobacter sakazakii* [27], clustered with *E. cloacae* subsp. *cloacae* NCTC 9394 (0.90 posterior probability), which was isolated from human feces. Interestingly, all the *E. cloacae* strains did not cluster together.

**Table 1:** Classification and general features of *Enterobacter cloacae* EcWSU1 according to MIGS recommendations [9]

**MIGS ID**	**Property**	**Term**	**Evidence Code**
	Current classification	Domain *Bacteria*	TAS [10]
		Phylum *Proteobacteria*	TAS [11]
		Class *Gammaproteobacteria*	TAS [12,13]
		Order “*Enterobacteriales”*	TAS [14]
		Family *Enterobacteriaceae*	TAS [15-17]
		Genus *Enterobacter*	TAS [15,18-21]
		Species *Enterobacter cloacae*	TAS [15,18,21]
		Strain EcWSU1	TAS [8]
	Gram stain	negative	TAS [22]
	Cell shape	rod	TAS [22]
	Motility	motile via peritrichous flagella	TAS [22]
	Sporulation	non-sporulating	TAS [22]
	Temperature range	mesophilic, 25-40°C	TAS [22]
	Optimum temperature	30-37°C	TAS [22]
	Salinity	not reported	
MIGS-22	Oxygen requirement	facultative anaerobe	TAS [22]
	Carbon source	carbohydrates	TAS [22]
	Energy source	chemoorganotroph	TAS [22]
MIGS-6	Habitat	soil, onion	TAS [8]
MIGS-15	Biotic relationship	free-living	TAS [22]
MIGS-14	Pathogenicity	pathogenic on onion	TAS [8]
	Biosafety level	2	
	Isolation	Isolated from symptomatic onion	TAS [8]
MIGS-4	Geographic location	Colorado, USA	TAS [8]
MIGS-5	Sample collection time	not reported	

Evidence codes – IDA: Inferred from Direct Assay (first time in publication); TAS: Traceable Author Statement (i.e., a direct report exists in the literature); NAS: Non-traceable Author Statement (i.e., not directly observed for the living, isolated sample, but based on a generally accepted property for the species, or anecdotal evidence). These evidence codes are from the Gene Ontology project [23]. If the evidence code is IDA, then the property was directly observed for a live isolate by one of the authors or an expert mentioned in the acknowledgements.

## Genome project history

### Genome sequencing and annotation

*E. cloacae* EcWSU1 was isolated from onions exhibiting symptoms of *Enterobacter* bulb decay [8]. EcWSU1 is the model strain for studying pathogenesis of *E. cloacae* on onion in the laboratory of Brenda Schroeder at Washington State University. A genome sequence of EcWSU1 was needed to facilitate the development of molecular biology experiments. Pyrosequencing of EcWSU1 was completed at the Laboratory for Biotechnology and Bioanalysis at Washington State University, and the PCR products to close the genome were sequenced at Elim Biopharmaceuticals (Hayward, CA, USA). The complete chromosome sequence as well as the mega-plasmid, pEcWSU1_A, has been deposited in Genbank under the accession numbers CP002886 and CP002887, respectively. Table 2 summarizes the EcWSU1 sequencing project.

**Table 2:** EcWSU1 Genome sequencing project information

**MIGS ID**	**Property**	**Term**
MIGS-31	Finishing quality	Finished
MIGS-29	Sequencing platform	454 Life Sciences
MIGS-31.2	Fold coverage	20 ×
MIGS-30	Assembler	GS De novo Assembler V2.3
MIGS-32	Gene calling method	Bacterial Annotation System (BASys) [28]
		tRNAscan-SE 1.21 [29]
	Genbank ID	CP002886 (chromosome)
		CP002887 (pEcWSU1_A)
	Genbank date of release	With SIGS publication
	Project relevance	Plant pathology

### Growth conditions and DNA isolation

*E. cloacae* EcWSU1 was cultured overnight in 5 ml of LB broth [30] in a 20 ml glass culture tube (16 mm O.D.) on a rotary shaker at 200 rpm at 28°C. Prior to genomic DNA isolation, the cells were washed twice with equal volumes of sterile, distilled water to remove excess exopolysaccharides. Genomic DNA was then isolated from the washed cells using a Wizard Genomic DNA Purification Kit (Promega, A1120) following the kit protocol for Gram-negative bacteria.

### Genome sequencing and assembly

The genomic DNA extraction showed a high absorbance at 230 nm during quantification, indicating the presence of polysaccharides. As a result, prior to preparing the DNA for pyrosequencing, the polysaccharides were selectively precipitated in 20% ethanol and removed from the sample by centrifugation. The DNA was then precipitated with two volumes of ethanol, pelleted via centrifugation, dried and suspended in TE buffer (10 mM Tris, 1 mM EDTA, pH 8). The sequencing library was constructed using 500 ng of the genomic DNA with the GS FLX Titanium Rapid Library Preparation Kit (Roche, 05608228001) and RL MID adapters (Roche, 05619211001) in place of the standard RL adapters. Minor modifications to the protocol included more extensive washing at the sequencing bead enrichment and harvest steps. The resulting shotgun library was diluted 1:5 and 10 µl was quantified using a 384-well fluorescent plate assay in a Perkin-Elmer Victor X Multi-label Plate Reader. Quality and size of the library was assessed using an Agilent High Sensitivity DNA chip assay (Agilent, 5067-4626) read on an Agilent 2100 Bioanalyzer. Pyrosequencing was performed on a Genome Sequencer GS FLX Titanium instrument (454 Life Sciences, Branford, CT, USA) with the sample occupying one quarter of one picotiter plate. A total of 242,000 reads were obtained accounting for 97.5 Mb of sequence. Reads were assembled using GS De Novo Assembler V2.3 with default parameters and 99.7% of the bases aligned into 35 contigs with 27 of those greater than 5 kb. For the contigs, 281 bp remained at 1× coverage with a bimodal peak depth predominantly centered at 20× that trailed into a second smaller peak of coverage at 141-180× resulting from a higher plasmid copy number relative to genomic DNA in the sample.

The genome sequence of *E. cloacae* subsp. *cloacae* ATCC 13047 (CP001918) initially was used as a reference sequence for assembly of the pyrosequencing reads. However, the genomic sequence of EcWSU1 did not have sufficient identity to the DNA sequence of ATCC 13047 for this to be effective (only 19.56% of the reads mapped to ATCC 13047). As a result, the EcWSU1 genome was closed by developing primers that amplified out from each end of the contigs. A putative contig order was generated by using blastn to align the 35 contigs against the incomplete genome (18 contigs) of *E. cloacae* P101 [31-33], an endophyte of switchgrass that had higher DNA similarity to EcWSU1 than EcWSU1 had with ATCC 13047. The putative contig order of EcWSU1 was then confirmed with PCR amplifications across the contig junctions using GoTaq Polymerase (Promega, M3001) according to the manufacturer’s protocol and 50 ng of EcWSU1 genomic DNA. An annealing temperature of 52°C, with an extension of 1 m was sufficient for most of the contig junctions since there usually were 0-50 bases missing between the contigs. DMSO was added at either a 5% or 10% final concentration in the PCR reaction, in combination with an extension time of 8.5 m, to produce larger fragments that amplified across the 16S-23S rRNA cassettes or to amplify contig junctions that would not amplify with the normal PCR reaction used above. Sequencing was completed for both strands using the same primers used for amplification of the fragments. Fragments that spanned the 16S-23S rRNA regions were also sequenced with internal primers that were specific for contigs that corresponded to the 16S and 23S rRNA regions of EcWSU1. The contigs and sequences from the PCR products were aligned with Bioedit (Ibis Biosciences, Carlsbad, CA) and a complete chromosome sequence was generated with 34 of the 35 contigs. The remaining contig of 63.7 kb was shown to be circular and was designated as pEcWSU1_A.

### Genome annotation

Genome annotation was completed using the Bacterial Annotation System (BASys) [28]. tRNA sequences were determined using tRNAscan-SE [29] and rRNA sequences were identified by searching the genome sequence with rRNA sequences from *E. cloacae* subsp. *cloacae* ATCC 13047 using a private nucleotide BLAST server [34]. Minor editing to the annotation to remove ORFs that were completely contained in other ORFs was done, and the features file was generated using in-house Java programs. The submission file for Genbank was prepared using Sequin from the NCBI website.

## Genome properties

The genome of *E. cloacae* EcWSU1 consists of one circular chromosome of 4,734,438 bp and a mega-plasmid, pEcWSU1_A, of 63,653 bp. The average G+C content for the genome is 54.5% (Table 3). There are 83 tRNA genes and 8 rRNA operons each consisting of a 16S, 23S, and 5S rRNA gene. There are 4,632 predicted protein-coding regions and 13 pseudogenes in the genome. A total of 4,122 genes (87.0%) have been assigned a predicted function while the rest have been designated as hypothetical proteins (Table 3). The numbers of genes assigned to each COG functional category are listed in Table 4. About one sixth (15.3%) of the annotated genes were not assigned to a COG or have an unknown function.

**Table 3:** EcWSU1 Genome Statistics

**Attribute**	**Value**	**% of total^a^**
Genome size (bp)	4,798,091	100%
DNA coding region (bp)	4,326,148	90.16%
DNA G+C content (bp)	2,616,970	54.54%
Number of replicons	2	
Extrachromosomal elements	1	
Total genes^b^	4,740	100%
tRNA genes	83	1.75%
rRNA operons	8	
Protein-coding regions	4,632	97.72%
Pseudo genes	13	0.27%
Genes with function prediction	4,122	86.96%
Genes in paralog clusters	322	7.00%
Genes assigned to COGs	3,830	80.80%
Genes assigned Pfam domains	3,972	83.80%
Genes with signal peptides	898	18.95%
Genes with transmembrane helices	1143	24.11%
CRISPR repeats	0	

^a^ The total is based on either the total number of base pairs or the total number of genes in the genome and includes the chromosome and the plasmid pEcWSU1_A

^b^ Includes the tRNA genes and pseudogenes

**Table 4:** Number of genes associated with the general COG functional categories

Code	Value	% age	Description
J	190	4.1	Translation, ribosomal structure and biogenesis
A	1	0.0	RNA processing and modification
K	401	8.7	Transcription
L	147	3.2	Replication, recombination and repair
B	0	0.0	Chromatin structure and dynamics
D	33	0.7	Cell cycle control, cell division, chromosome partitioning
Y	0	0.0	Nuclear structure
V	53	1.1	Defense mechanisms
T	221	4.8	Signal transduction mechanisms
M	248	5.4	Cell wall/membrane/envelope biogenesis
N	109	2.4	Cell motility
Z	0	0.0	Cytoskeleton
W	0	0.0	Extracellular structures
U	113	2.4	Intracellular trafficking, secretion, and vesicular transport
O	145	3.1	Posttranslational modification, protein turnover, chaperones
C	235	5.1	Energy production and conversion
G	455	9.8	Carbohydrate transport and metabolism
E	385	8.3	Amino acid transport and metabolism
F	88	1.9	Nucleotide transport and metabolism
H	169	3.6	Coenzyme transport and metabolism
I	124	2.7	Lipid transport and metabolism
P	232	5.0	Inorganic ion transport and metabolism
Q	91	2.0	Secondary metabolites biosynthesis, transport and catabolism
R	483	1.0	General function prediction only
S	366	7.9	Function unknown
-	343	7.4	Not in COGs

^a^ The total is based on the total number of protein coding genes in the entire annotated genome
